# Biosynthesis and Characterization of Selenium Nanoparticles by *Kocuria* Strain Bm3: Evaluating Their Synergistic Antimicrobial Activity with *Chlorella* *vulgaris* Crude Extract Against Multidrug-Resistant Bacteria

**DOI:** 10.5812/ijpr-164350

**Published:** 2025-11-30

**Authors:** Hamid Babavalian, Fatemeh Shakeri, Atefeh Safarpour, Iman Hassankhani, Zohre Nasrollahzadeh, Mohammad Ali Amoozegar

**Affiliations:** 1 Applied Biotechnology Research Center, New Health Technologies Institute, Baqiyatallah University of Medical Sciences, Tehran, Iran; 2 Extremophiles Laboratory, Department of Microbiology, School of Biology and Center of Excellence in Phylogeny of Living Organisms, College of Science, University of Tehran, Tehran, Iran

**Keywords:** Selenium Nanoparticles, *Kocuria*, *Chlorella Vulgaris*, Antimicrobial Activity, Bioremediation

## Abstract

**Background:**

Mangrove ecosystems are rich in halotolerant microorganisms capable of synthesizing antimicrobial nanoparticles. Exploring these microorganisms for the development of novel antimicrobial agents is increasingly important, particularly given the escalating problem of multidrug resistance.

**Objectives:**

This study aimed to confirm the biosynthesis of selenium nanoparticles (SeNPs) by the *Kocuria* strain Bm3, isolated from the Hera mangroves in Iran, and to evaluate the antimicrobial efficacy of SeNPs in combination with *Chlorella vulgaris* crude extract (*C. vulgaris* CE) against various bacterial strains.

**Methods:**

The synthesized SeNPs were characterized with respect to morphology and size. Antimicrobial activity was assessed by measuring inhibition zones; minimum inhibitory concentration (MIC) and minimum bactericidal concentration (MBC) values were determined. Synergistic effects were calculated using the Fractional Inhibitory Concentration Index (ΣFIC). Additionally, cytotoxic effects on MCF-7 cell lines were evaluated.

**Results:**

The combination of *C. vulgaris* CE and SeNPs significantly enhanced antibacterial activity against various strains, most notably a clinical isolate of *Escherichia coli*, which exhibited marked growth inhibition and reduced MIC values (P < 0.001). While *Staphylococcus aureus* did not demonstrate significant differences among treatments, *Staphylococcus epidermidis* and *Staphylococcus saprophyticus* showed improved sensitivity with the combination treatment, achieving substantial growth inhibition zones (P < 0.001). In contrast, the clinical isolate of *Acinetobacter baumannii* displayed potential resistance, with no growth inhibition observed when treated alone. The MTT assay revealed that *C. vulgaris* CE and SeNPs each exhibited low cytotoxicity individually, resulting in MCF-7 cell viability percentages of 49.75 ± 3.75% and 44.46 ± 4.26%, respectively; however, their combination significantly reduced cell viability to 29.36 ± 2.64% (P < 0.001).

**Conclusions:**

The SeNPs synthesized by the *Kocuria* strain Bm3 significantly enhance the antimicrobial properties of *C. vulgaris* CE, indicating their potential as a therapeutic strategy against multidrug-resistant infections. Further investigation into their cytotoxic effects is warranted to evaluate safety and efficacy for clinical applications.

## 1. Background

Mangrove ecosystems, characterized by their unique biodiversity and capacity to thrive in saline environments, serve as critical interfaces between terrestrial and marine ecosystems. These ecosystems harbor a diverse array of halophilic and halotolerant microorganisms that have evolved specialized mechanisms enabling survival and proliferation under extreme salinity conditions (1). Among the various environmental stressors, heavy metal contamination — particularly from anthropogenic activities — poses a significant threat to these ecosystems. Microorganisms inhabiting mangrove habitats have developed tolerance mechanisms to cope with elevated metal concentrations, including selenium (Se), which functions as both an essential micronutrient and a toxic element at higher levels. Understanding the adaptive strategies of these microorganisms is essential for developing bioremediation approaches that leverage their intrinsic capabilities (2).

Bacteria possess a remarkable ability to synthesize selenium nanoparticles (SeNPs) via both intracellular and extracellular pathways. The biogenic synthesis of SeNPs offers several advantages over conventional chemical methods, such as lower toxicity, diminished environmental impact, and the potential for functionalization with bioactive compounds (3). The SeNPs possess unique physicochemical properties that impart antimicrobial, antioxidant, and anticancer activities, rendering them valuable for diverse biomedical applications. The capacity of certain bacterial strains to produce SeNPs in response to selenite exposure not only underscores their potential for bioremediation but also positions them as promising candidates for the development of novel therapeutic agents (4).

*Chlorella vulgaris*, a green microalga renowned for its rich content of bioactive compounds, has attracted attention for its potential health benefits, including antibacterial and antioxidant properties. Recent studies indicate that crude extracts derived from *C. vulgaris* can enhance the antimicrobial efficacy of nanoparticles, resulting in a synergistic effect that may help overcome the challenges posed by antibiotic resistance (5). The combination of *Chlorella vulgaris* crude extract (*C. vulgaris* CE) with SeNPs may provide a multifaceted strategy to combat pathogenic bacteria, particularly those exhibiting drug resistance. This underscores the importance of exploring such combinations in the pursuit of effective antimicrobial strategies (6).

*Chlorella vulgaris* extracts are abundant in various bioactive compounds, such as total phenolics, proteins, and chlorophyll, all of which contribute to their antioxidant, anti-inflammatory, and antimicrobial activities. Research demonstrates that *C. vulgaris* can comprise up to 50 - 60% protein by dry weight, making it a valuable nutritional supplement. The presence of total phenolics in *Chlorella* extracts correlates with enhanced antioxidant activity, potentially aiding in the mitigation of oxidative stress in different biological systems. Furthermore, chlorophyll, in addition to imparting color to the algae, exhibits therapeutic effects, including antibacterial and anticancer properties (7).

The selection of *C. vulgaris* CE for evaluating synergistic effects with SeNPs is especially justified due to its distinctive composition of bioactive compounds, which have shown promise in enhancing antimicrobial efficacy. The interaction between these bioactive compounds and nanoparticles may facilitate improved penetration and effectiveness against resistant bacterial strains. Moreover, the natural origin and established safety profile of *C. vulgaris* as a food supplement support its application in the development of innovative antimicrobial strategies (8).

## 2. Objectives

In this study, we isolated and characterized a selenite-tolerant bacterium from the Hera mangrove ecosystem in southern Iran, focusing on its ability to synthesize SeNPs through both intracellular and extracellular methods. We further investigated the antibacterial activity of the synthesized SeNPs in combination with *C. vulgaris* CE against various pathogenic bacteria, including clinical isolates resistant to conventional antibiotics. Additionally, we evaluated the cytotoxic effects of these formulations on the MCF-7 cell line to assess their safety for potential therapeutic applications. By integrating microbial biotechnology with natural product chemistry, this research aims to contribute valuable insights into sustainable bioremediation and innovative antimicrobial strategies.

## 3. Methods

### 3.1. Sample Collection and Isolation of Halotolerant/Halophilic Selenite-Tolerant Bacteria

Water samples were collected aseptically from the mangrove ecosystem in southern Iran (26°58'17.288"N, 55°37'49.694"E). The samples were stored in sterile polythene bags and transported to the laboratory for further analysis. Upon arrival, several physicochemical parameters of the water samples were evaluated, including temperature, pH, salinity, electrical conductivity, and color. The pH was measured under controlled conditions using a calibrated pH meter (Mettler Toledo, Singapore). Salinity was determined using a conductivity meter (Mettler Toledo, Singapore), and electrical conductivity was also assessed. The enrichment and isolation of halotolerant/halophilic bacteria were performed following the method described by Amoozegar et al. (9), with some modifications. A 50 mL aliquot of the collected sample was centrifuged at 4000 g for 20 minutes. The supernatant (45 mL) was discarded, and the remaining 5 mL was transferred to Erlenmeyer flasks containing 20 mL of MRM-3 (marine medium broth with a final total salt concentration of 3% w/v). The composition of MRM-3 (g L^-1^) included: NaCl (1.985), MgCl_2_·6H_2_O (0.88), MgSO4·7H_2_O (0.324), CaCl_2_ (0.18), KCl (0.055), NaHCO_3_ (0.016), yeast extract (1), and peptone from meat (5). The flasks were then incubated under orbital shaking at 150 rpm and 37°C for 2 days. After incubation, enrichment cultures exhibiting turbidity were subcultured on MRM-3 agar and incubated at 37°C for an additional 2 days. Morphologically distinct bacterial colonies were selected and streaked on the same solid medium at least three times to obtain pure cultures. Pure halotolerant/halophilic bacterial colonies were subsequently cultured on SW-3 (sea water medium containing a total salt concentration of 3% w/v), which consisted of: NaCl (1.985), MgCl_2_·6H_2_O (0.88), MgSO_4_·7H_2_O (0.324), CaCl_2_ (0.18), KCl (0.055), NaHCO_3_ (0.016), yeast extract (1), and agar (10). This medium was amended with 3 mM of sodium selenite (11). Following this, selenite-tolerant bacteria were subjected to further investigations.

### 3.2. Morphological, Biochemical, Physiological, and Molecular Characterization of the Selenite-Tolerant Bacterium

The morphological characteristics of the selected selenite-tolerant bacterial isolate were examined, focusing on colony size, shape, margin, elevation, chromogenesis, surface texture, and optical properties. For biochemical characterization, a series of standard tests were performed after incubating the isolate for 24 hours at 37°C on MRM-3 agar. These tests included Gram-staining, oxidase test, catalase test, and motility assessment. Physiological studies were conducted to evaluate bacterial growth under varying conditions in MRM-3 medium. The sensitivity of the isolate to different salinity levels (0 - 23% w/v), temperatures (20 - 45°C), and pH ranges (5.0 - 8.0) was assessed. For molecular characterization, genomic DNA was extracted using the following method: The peptidoglycan-rich cell wall was first digested with lysozyme (10 mg/mL) to facilitate the breakdown of cell wall components. A 10% (w/v) sodium dodecyl sulfate (SDS) solution was then used to disrupt cell membranes and denature proteins. Additionally, proteinase K was employed to effectively digest various proteins within the cell membrane and cytoplasm, including DNases (12). The quality of the extracted genomic DNA was confirmed using 1% (w/v) agarose gel electrophoresis. The bacterial 16S rRNA gene was amplified using the extracted genomic DNA as the template. The PCR reaction included 10 ng of genomic DNA and utilized universal bacterial primers 27F (5´-AGAGTTTGATCCTGGCTCAG-3') and 1492R (5´-GGTTACCTTGTTACGACTT-3'). The amplification was carried out in a 30 μL reaction mixture containing 0.2 mM of each deoxynucleoside triphosphate (dNTP), 1 μM of primers 27F and 1492R, and one unit of Taq DNA polymerase with its supplied 1× buffer (Fermentas, Hanover, Germany). The PCR program consisted of an initial denaturation at 95°C for 5 minutes, followed by 30 cycles of denaturation at 94°C for 1 minute, primer annealing at 60°C for 1 minute, extension at 72°C for 1 minute, and a final extension at 72°C for 10 minutes. The PCR products were analyzed on a 1% agarose gel and subsequently sent to Macrogen Inc. (South Korea) for sequencing. The obtained sequences were identified based on similarities with known 16S rRNA gene sequences using the EzBioCloud web server (13). The retrieved data were aligned using ClustalX2, and a phylogenetic tree was constructed using the neighbor-joining algorithm with the Tamura-Nei model in MEGA 11 software. Bootstrap analysis with 1000 bp replicates was performed to assess the confidence level of the phylogenetic tree. The 16S rRNA gene sequence obtained in this study has been deposited in the NCBI GenBank under accession number PV776680. The methods and results for the synthesis and characterization of SeNPs from the strain are detailed in the Supplementary File, along with the Supplementary Figures 1 and 2. 

### 3.3. Isolation, Cultivation and Preparation of Chlorella vulgaris Crude Extract

Microalgae *C. vulgaris* strain sh4 (IBRC-M50026) were obtained from the Iranian Biological Resource Center in Tehran, Iran. The microalgae were cultured on Blue-Green Medium (BG-11) agar using a lawn culture technique. The BG-11 medium was prepared with the following components (g L^-1^): NaNO_3_ (1.5), K_2_HPO_4_ (0.04), MgSO_4_·7H_2_O (0.075), CaCl_2_·2H_2_O (0.036), citric acid (0.006), ferric ammonium citrate (0.006), EDTA (disodium salt) (0.001), Na_2_CO_3_ (0.02), and agar (if required) (10.0), supplemented with 1.0 mL of Trace Metal Mix A5. The trace metal mix was prepared with the following components (g L^-1^): H_3_BO_3_ (2.86), MnCl_2_·4H_2_O (1.81), ZnSO_4_·7H_2_O (0.222), NaMoO_4_·2H_2_O (0.39), CuSO_4_·5H_2_O (0.079), and Co(NO_3_)_2_·6H_2_O (49.41 × 10^-3^). The cultures were maintained under stable conditions at a temperature of 25°C and an appropriate light intensity of 37 µmol m^-2^ s^-1^. Once isolated colonies formed on the solid medium, one colony was transferred to BG-11 broth in 1000 µL vials and incubated under the same light and temperature conditions. Over a period of 15 days, the volume of the liquid medium was gradually increased until it reached a final volume of 500 mL. This final volume was then used for culturing microalgae in larger volumes (5 - 6 L) across multiple containers. The biomass was harvested via centrifugation at 6000 rpm for 6 minutes using an MPW-350R centrifuge (Germany).

Following centrifugation, a mixture of chloroform and methanol in a 2:1 ratio was added to the biomass at a ratio of 5 grams per 200 mL. This mixture was then incubated at 40°C for 48 hours to initiate the extraction. After incubation, soluble compounds were separated from solid residues through filtration. Next, 200 µg/mL of Alcalase enzyme (Sigma, USA) was introduced to the algal solution for enzymatic treatment, and the mixture was incubated at 50°C for 30 minutes to facilitate enzymatic hydrolysis. The crude extract was then centrifuged at 13,000 rpm for 20 minutes to separate cellular solid particles. The resulting supernatant was filtered through a 0.22-micron membrane filter and concentrated using a rotary evaporator (R206B 2L, SENCO China) at a temperature of 45°C to yield the *C. vulgaris* CE (14).

### 3.4. Well Diffusion Assay of Selenium Nanoparticles and Chlorella vulgaris Crude Extract Against Pathogenic Bacteria

Pathogenic bacteria were obtained from the Iranian Biological Resource Center, Tehran, Iran, including *Escherichia coli* (IBRC-M10208), *Staphylococcus aureus* (IBRC-M10690), *Acinetobacter baumannii* (IBRC-M10654), *S. epidermidis* (IBRC-M10694), and *S. saprophyticus* (IBRC-M10635). Additionally, three drug-resistant pathogenic bacteria were sourced from a clinical microbiology laboratory of Baqiyatallah Hospital (Tehran, Iran), including clinical *E. coli*, clinical *A. baumannii*, and clinical *S. aureus* sp. A+. These strains were maintained on nutrient agar slants at 4°C until further use.

The antibacterial activity of SeNPs, *C. vulgaris* CE, and their combination was evaluated using the well diffusion assay. Nutrient agar plates were prepared and inoculated with the pathogenic bacterial strains to achieve a uniform lawn of growth. Wells (approximately 6 mm in diameter) were created in the agar using a sterile cork borer. Solutions of SeNPs, *C. vulgaris* CE, and a combination of both were prepared at a concentration of 500 mg/L in a mixture of 80% sterile distilled water with 20% dimethyl sulfoxide (DMSO). Each well was filled with 100 µL of the respective treatment solution.

Negative control wells contained only the mixture of 80% sterile distilled water with 20% DMSO to assess any potential effects of the solvent on bacterial growth. No significant changes were observed (data not shown). Positive control wells were filled with ampicillin antibiotic at a concentration of 100 µg/mL (15). The plates were incubated at 37°C for 24 hours. After incubation, the diameter of the inhibition zones surrounding each well was measured in millimeters. The results were recorded, and the antibacterial activity of SeNPs, *C. vulgaris* CE, and the combination of SeNPs plus *C. vulgaris* CE against the tested bacterial strains was evaluated based on the size of the inhibition zones.

### 3.5. Determination of the Minimum Inhibitory Concentration, Minimum Bactericidal Concentration, and Fractional Inhibitory Concentration of Selenium Nanoparticles and Chlorella vulgaris Crude Extract Against Pathogenic Bacteria

The minimum inhibitory concentration (MIC) of SeNPs, *C. vulgaris* CE, and their combination against pathogenic bacteria was determined using an agar dilution method. For this purpose, the bacterial isolates were subjected to serial dilutions of SeNPs (0 - 10,000 mg/L), *C. vulgaris* CE (0 - 10,000 mg/L), and the combination of SeNPs plus *C. vulgaris* CE (0 - 10,000 mg/L) in Mueller-Hinton broth. The cultures were incubated at 37°C with shaking at 150 rpm for 24 hours. Growth was monitored by measuring absorbance at 600 nm. Negative control groups consisted of bacterial cultures devoid of the respective solvents of SeNPs and *C. vulgaris* CE. The lowest concentration of SeNPs, *C. vulgaris* CE, or their combination that inhibited bacterial growth was considered the MIC value.

For the determination of the minimum bactericidal concentration (MBC), 10 µL of each MIC replicate was cultured on Mueller-Hinton agar, and the lowest concentration that resulted in no visible growth after incubation was selected as the MBC value. The fractional inhibitory concentration (FIC) for each agent was calculated using the following formula: FIC=(MIC of combination/MIC of individual agent). For SeNPs and *C. vulgaris* CE, this calculation was performed separately for each bacterial strain. The Fractional Inhibitory Concentration Index (ΣFIC) was determined by summing the individual FIC values for both agents: ΣFIC=[FIC (SeNPs)+FIC (*C. vulgaris* CE)] (16).

### 3.6. Evaluation of Cytotoxic Effects of Selenium Nanoparticles and Chlorella vulgaris Crude Extract on MCF-7 Cell Line

The MCF-7 cell line (IBRC C10082) was obtained from the Iranian Biological Resource Center, Tehran, Iran. The cells were cultured in DMEM high glucose medium (GIBCO, Germany), supplemented with 10% (v/v) fetal bovine serum (FBS), 1.0 mM sodium pyruvate, 2 mM L-glutamine, 100 U/mL penicillin, and 100 μg/mL streptomycin (all from GIBCO, Germany). Cultures were maintained at 37°C in a humidified atmosphere containing 95% air and 5% CO_2_.

The effects of SeNPs at a concentration of 500 mg/L, *C. vulgaris* CE at the same concentration, and their combination (5 mg/L) on cell viability were assessed using the MTT assay, as established in previous studies (17-19). Solutions were prepared in a mixture of 80% sterile distilled water with 20% DMSO. Approximately 5,000 cells were seeded in each well of a 96-well plate and allowed to adhere for 24 hours. Following this, the cells were treated with SeNPs, *C. vulgaris* CE, or their combination for 48 hours. The effects of the solvent (mixture of 80% sterile distilled water with 20% DMSO) were also evaluated in the same manner as a negative control, with no significant changes observed (data not shown).

After treatment, 10 μL of a 5 mg/mL MTT solution in PBS was added to each well, and the plates were incubated for an additional 3 hours at 37°C. Subsequently, the medium was removed, and 100 μL of DMSO was added to each well to dissolve the blue formazan crystals formed during the assay. The absorbance was measured at 560 nm using a microplate reader. One well of each plate containing only DMSO was used as a blank control. The optical density (OD) values for both control and treated groups were adjusted by subtracting the blank OD from each well's reading. The percentage of viable cells was calculated using the following formula: Percentage of viable cells=([mean OD (treated)]/[mean OD (control)])×100 (20).

### 3.7. Statistical Analysis

All experiments, including well diffusion assays, MTT assays, MIC/MBC determinations, and FIC tests, were conducted with four biological replicates. The MTT and MIC assays included three technical replicates for each biological replicate. Data are expressed as means ± standard deviation (SD). Differences between control and treatment groups were analyzed using one-way ANOVA, followed by independent samples *t*-tests where appropriate, with P < 0.05 considered statistically significant.

## 4. Results

### 4.1. Isolation, Characterization and Identification of Selenite-Tolerant Bacterial Strain Bm3

**Figure 1.**
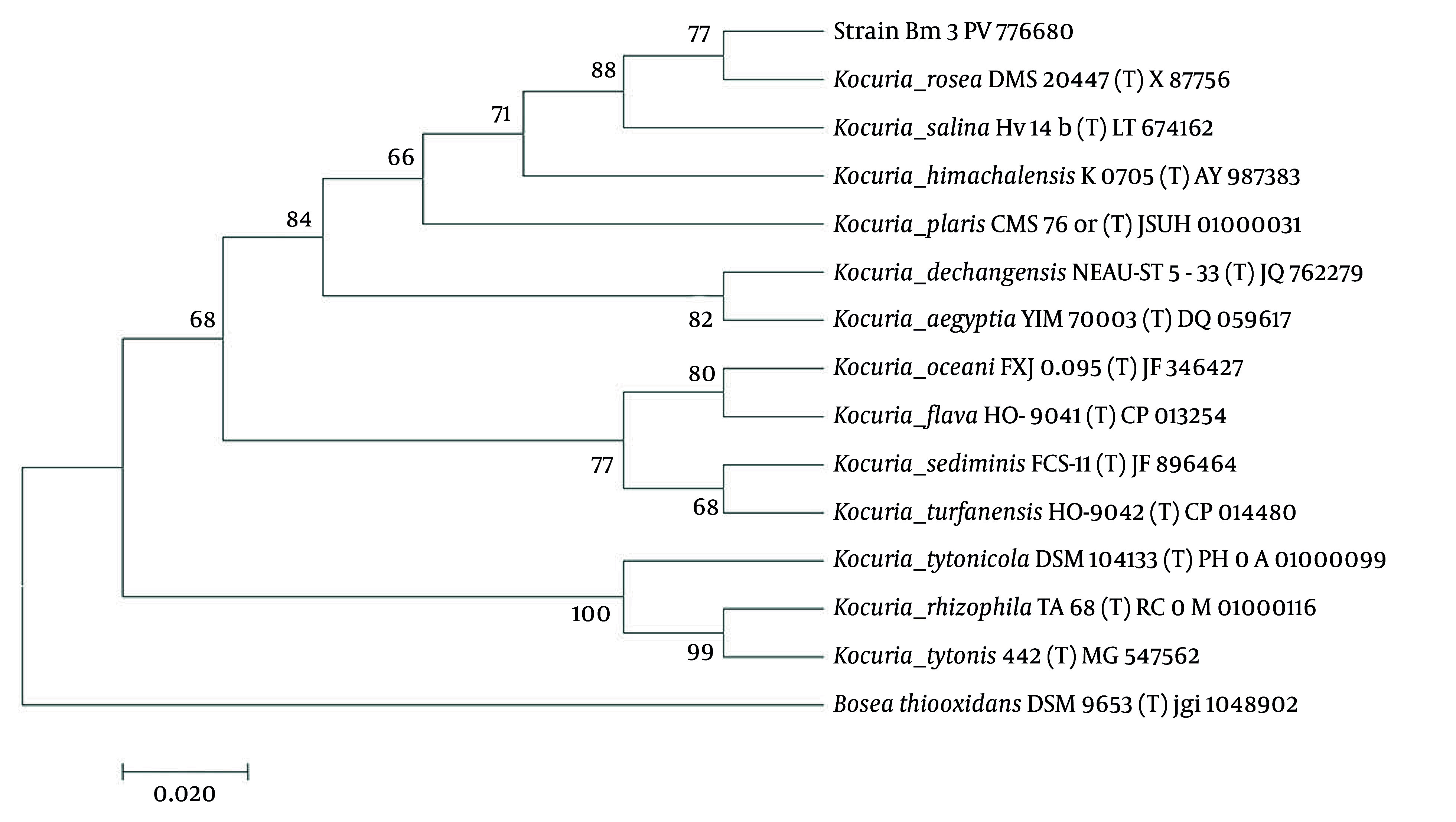
Phylogenetic tree based on 16S rRNA gene sequences: The phylogenetic tree shows the position of strain Bm3 among other strains of the genus *Kocuria* (bootstrap values are based on 1000 replicates).

Water samples were aseptically collected from the Hera mangrove ecosystem in southern Iran at a depth of 10 - 20 cm. The physicochemical analysis of the water revealed a temperature of 30.8°C, salinity of 4%, and pH of 7.6. Strains isolated from this environment were cultured on MRM-3 medium, followed by an assessment of their resistance to sodium selenite using SW-3 medium. This screening process identified one strain, designated Bm3. Biochemical characterization of the sodium selenite-tolerant strain Bm3 revealed significant traits, including positive salt tolerance at NaCl concentrations ranging from 0% to 5%, with optimal growth observed at 2%, classifying it as halotolerant. Gram-staining indicated that strain Bm3 is a gram-positive coccus and is non-spore-forming. Colony morphology was characterized by slightly raised, glossy, light pink colonies that were small, circular, with well-defined margins and a smooth texture. Further biochemical tests confirmed the identity of strain Bm3, which tested positive for catalase, oxidase, and the KOH test. The taxonomic identity of this halotolerant bacterial isolate was confirmed through analysis of the 16S rRNA gene sequences (1,031 bp) using EzBioCloud, revealing 99.32% homology with *Kocuria* *rosea*. A neighbor-joining phylogenetic tree illustrating the relationship of strain Bm3 (hereafter referred to as *Kocuria* strain Bm3) to related taxa is presented in Figure 1. 

### 4.2. Antimicrobial Properties and Cytotoxicity of Selenium Nanoparticles and Chlorella vulgaris Crude Extract

The antimicrobial activity of SeNPs in combination with *C. vulgaris* CE was evaluated against various bacterial strains, including clinical isolates and pathogenic bacteria. Inhibitory growth zone measurements revealed variable antimicrobial effects of SeNPs, *C. vulgaris* CE, and their combination at a concentration of 500 mg/L. For *E. coli*, the combination of *C. vulgaris* CE and SeNPs significantly enhanced the inhibitory effect compared to *C. vulgaris* CE alone, increasing the growth inhibition zone from 32 ± 1 mm to 39 ± 1 mm (P < 0.001). However, no significant difference was observed between SeNPs alone and their combination for this strain. In the case of a clinical isolate of *E. coli*, the combination significantly increased the growth inhibition zone to 12 ± 1 mm (P < 0.001). Similarly, for *A. baumannii*, the growth inhibition zones significantly increased from 35 ± 1 mm for SeNPs and 38 ± 1 mm for *C. vulgaris* CE to 42 ± 1 mm when combined (P < 0.001 and P < 0.01, respectively). Notably, *S. aureus* did not show a significant increase in the inhibition zone among the treatment groups. Conversely, the clinical isolate of *A. baumannii* exhibited no growth inhibition when tested alone, indicating potential resistance to the treatments. For *S. epidermidis*, results were mixed; there was no inhibition with SeNPs alone, but a significant increase to 29 ± 1 mm was observed when combined with *C. vulgaris* CE (P < 0.001). Additionally, a significant increase in the inhibition zone was noted for *S. epidermidis*, rising from 25 ± 1 mm with *C. vulgaris* CE to a greater value when combined with SeNPs (P < 0.01). Furthermore, *S. saprophyticus* demonstrated improved sensitivity to both SeNPs and *C. vulgaris* CE, with inhibition zones measuring 36 ± 1 mm and 42 ± 1 mm, respectively, and significantly reaching 47 ± 1 mm in their combination (P < 0.001).

**Figure 2.**
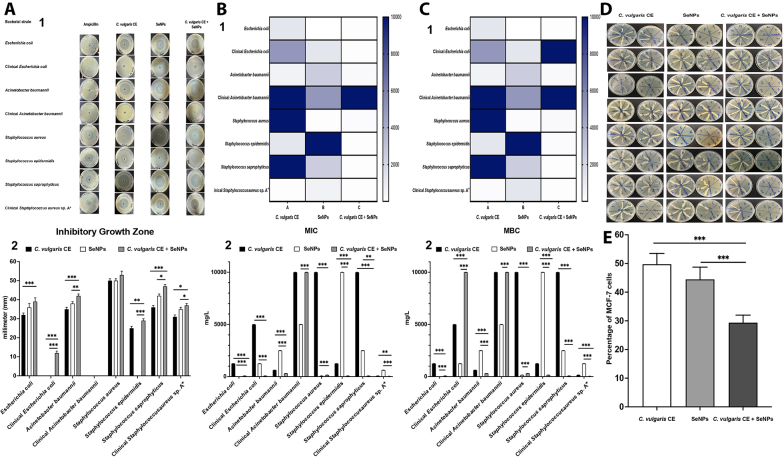
Antimicrobial properties and cytotoxicity of selenium nanoparticles (SeNPs) and *Chlorella vulgaris* crude extract (*C. vulgaris* CE). A, well diffusion assay results demonstrating the antimicrobial activity of SeNPs and *C. vulgaris* CE against various pathogenic bacteria: (1) Growth inhibition zones were measured in millimeters (mm) for each treatment combination, including SeNPs, *C. vulgaris* CE, their combination (SeNPs + *C. vulgaris* CE), and ampicillin (100 µg/mL) against *Escherichia coli*, clinical *E. coli*, *Acinetobacter baumannii*, clinical *A. baumannii*, *Staphylococcus aureus*, *S. epidermidis*, *S. saprophyticus*, and clinical *S. aureus* sp. A+. (2) A bar chart illustrating the results of the well diffusion assay of SeNPs, *C. vulgaris* CE, and their combination against pathogenic bacteria; B, minimum inhibitory concentration (MIC) and; C, minimum bactericidal concentration (MBC) data for the aforementioned bacterial strains in the presence of SeNPs, *C. vulgaris* CE, and their combination (SeNPs + *C. vulgaris* CE). The MIC and MBC data are presented as (1) heatmap and (2) bar chart; D, MBC results for SeNPs and *C. vulgaris* CE against pathogenic bacteria, with growth inhibition assessed across different concentrations (0 - 10,000 mg/L) of SeNPs, *C. vulgaris* CE, and their combination (SeNPs + *C. vulgaris* CE); E, cytotoxicity assessment using the MTT assay on the MCF-7 cell line for SeNPs, *C. vulgaris* CE, and their combination, with significance indicated as follows: * P < 0.05, ** P < 0.01, and *** P < 0.001.

Ampicillin (100 µg/mL) (15) was used as a positive control, demonstrating inhibitory effects on various bacterial strains. The results showed that *E. coli* exhibited a zone of inhibition measuring 12 ± 1 mm, while *S. aureus* showed a larger zone of 40 ± 1 mm. Other strains, including *S. saprophyticus*, *S. epidermidis*, and clinical *S. aureus* sp. A+, displayed zones of inhibition measuring 27 ± 1 mm, 35 ± 1 mm, and 22 ± 1 mm, respectively. In contrast, the clinical strain of *E. coli* and both clinical and non-clinical strains of *A. baumannii* were found to be resistant (Figure 2A). 

As detailed Table 1 in the Supplementary File and Figure 2B and C, the antibacterial efficacy of the bacterial strains was further assessed using MIC, MBC, and FIC values. *Escherichia coli* showed a significant decrease in MIC and MBC values when treated with the combination of *C. vulgaris* CE and SeNPs compared to *C. vulgaris* CE alone (P < 0.001). In contrast, these values significantly increased in the combination treatment compared to SeNPs alone (P < 0.001), resulting in a ΣFIC value of 8.1036, indicating that SeNPs alone are more effective for this strain.

Conversely, the clinical isolate of *E. coli* exhibited significant synergy, with MIC values decreasing to 78 ± 1 mg/L (P < 0.001) when treated with the combination of *C. vulgaris* CE and SeNPs, compared to values of 5000 ± 1 mg/L and 1250 ± 1 mg/L for the individual treatments, respectively. This resulted in ΣFIC values of 0.0780, signifying synergy. Similarly, *A. baumannii* showed increased sensitivity (P < 0.001), with MIC and MBC values of 312 ± 1 mg/L when treated with the combination, whereas individual treatments with *C. vulgaris* CE and SeNPs yielded significantly higher values of 625 ± 1 mg/L and 2500 ± 1 mg/L, respectively (ΣFIC = 0.6240). For the clinical isolate of *A. baumannii*, the combination with SeNPs did not enhance the effect of *C. vulgaris* CE; however, it did lead to increased MIC and MBC values compared to SeNPs alone (P < 0.001), resulting in a ΣFIC of 3.0000.

Among gram-positive bacteria, *S. aureus* exhibited a significant decrease in MIC and MBC values with the combination treatment compared to *C. vulgaris* CE alone (P < 0.001). However, these values increased significantly in the combination compared to SeNPs alone (P < 0.001), leading to a ΣFIC value of 2.0156, indicating that SeNPs alone are more effective for this strain. *Staphylococcus* *epidermidis* also showed increased sensitivity (P < 0.001), with MIC and MBC values of 78 ± 1 mg/L and 156 ± 1 mg/L when treated with the combination, while individual treatments with *C. vulgaris* CE and SeNPs yielded significantly higher values (ΣFIC = 0.0702). *Staphylococcus* *saprophyticus* demonstrated reduced MIC and MBC values of 78 ± 1 mg/L when treated with the combination of SeNPs and *C. vulgaris* CE, compared to using them alone (P < 0.01 and P < 0.001, respectively; ΣFIC = 0.0390). Finally, the clinical isolate *S. aureus* sp. A+ exhibited reduced MBC values of 39 ± 1 mg/L (P < 0.001) when treated with the combination, compared to higher values for SeNPs and *C. vulgaris* CE alone. The observed results in MIC values for the combination were significantly lower compared to SeNPs (P < 0.001) and *C. vulgaris* CE alone (P < 0.01), with a ΣFIC value of 0.5624. The detailed MBC results are illustrated in Figure 2D. 

The data did not indicate a clear relationship between sensitivity to ampicillin and the observed synergistic effects in the strains tested. Ampicillin-sensitive strains, such as *E. coli* and *S. aureus*, showed a favorable response to SeNPs, exhibiting lower MIC and MBC concentrations. Additionally, other sensitive strains, including *S. saprophyticus*, *S. epidermidis*, and clinical *S. aureus* sp. A+, demonstrated a significant synergistic effect. In contrast, ampicillin-resistant strains, including the clinical strain of *E. coli* and the clinical strain of *A. baumannii*, did not exhibit any synergistic effects. However, another ampicillin-resistant strain, *A. baumannii*, displayed notable synergistic effects.

The viability of MCF-7 cell lines was assessed using the MTT assay to evaluate the cytotoxic effects of *C. vulgaris* CE, SeNPs, and their combination at a concentration of 5 mg/L (Figure 2E). The results indicated that both *C. vulgaris* CE and SeNPs exhibited relatively low cell viability, with percentages of 49.75 ± 3.75% and 44.46 ± 4.26%, respectively, suggesting low cytotoxicity when administered individually. However, the combination of *C. vulgaris* CE and SeNPs resulted in a significant decrease in cell viability, dropping to 29.36 ± 2.64% (P < 0.001).

## 5. Discussion

### 5.1. Synthesis and Characterization

*Kocuria* strain Bm3's resistance to sodium selenite underscores its adaptability and potential for bioremediation in saline environments such as mangroves. These findings are consistent with existing literature on halotolerant bacteria, which have been recognized for their ability to remediate heavy metal and metalloid pollution (10). By consolidating these insights, we highlight the broader implications of *Kocuria* species in bioremediation strategies (21). Furthermore, the documented capacity of *K. rosea* to produce nanoparticles adds another dimension to its biotechnological applications, suggesting that *Kocuria* strain Bm3 could be explored for similar nanoparticle synthesis, thereby enhancing its utility in environmental remediation efforts (22).

The differences in yield between intracellular and extracellular methods may significantly impact the biological activity of the synthesized SeNPs. Higher yields are typically associated with increased antimicrobial efficacy, as greater quantities of nanoparticles can lead to enhanced interactions with microbial cells (23). Future studies should investigate the relationship between yield and biological activity more comprehensively, potentially correlating specific yield thresholds with observable antimicrobial effects. Moreover, scalability is an important consideration for translating laboratory findings into practical applications. The current yields achieved through intracellular synthesis suggest a promising avenue for large-scale production; however, challenges such as maintaining optimal growth conditions and nutrient availability must be addressed (24). Future research should focus on optimizing growth parameters and exploring bioreactor systems capable of facilitating higher biomass and nanoparticle yields.

Characterization of *Kocuria* strain Bm3 through dynamic light scattering (DLS), Fourier-transform infrared spectroscopy (FTIR), X-ray diffraction (XRD), and scanning electron microscopy (SEM) confirms its capability to synthesize biogenic SeNPs with desirable properties. The SEM analysis reveals the presence of spherical nanoparticles with a uniform size distribution, which enhances antimicrobial activity due to increased surface area exposure. This observation aligns with findings by Sayed et al., indicating that spherical nanoparticles generally exhibit superior antimicrobial properties compared to irregularly shaped counterparts (23).

While we have characterized particle size and morphology, it is important to note that zeta potential measurements were not conducted in this study. Zeta potential is a critical parameter for assessing the stability and dispersion of nanoparticles in solution (25). Future stability testing methods utilizing UV-Vis spectroscopy should be explored to monitor changes in absorbance over time, which can indicate particle aggregation or degradation.

In summary, our findings demonstrate that *Kocuria* strain Bm3 is an effective candidate for biogenic SeNP synthesis, with implications for both environmental remediation and potential therapeutic applications.

### 5.2. Antimicrobial Activity and Synergy

The results of this study demonstrate the enhanced antimicrobial efficacy of the combination treatment of *C. vulgaris* CE and SeNPs against a variety of bacterial strains, particularly highlighting their synergistic effects against specific pathogens. These findings are especially significant for clinical isolates, which often exhibit resistance to conventional treatments (24). For instance, in the case of the clinical isolate of *E. coli*, the combination treatment markedly increased the growth inhibition zone compared to *C. vulgaris* CE and SeNPs alone, indicating a synergistic interaction that enhances the antimicrobial effect. The substantial increase in the inhibition zone suggests that *C. vulgaris* CE may potentiate the effects of SeNPs, thereby improving their overall efficacy (25). This is particularly relevant given the rising incidence of antibiotic-resistant *E. coli* strains in clinical settings.

However, it is noteworthy that the combination treatment yielded a significant reduction in MIC and MBC values for other strains, including *A. baumannii*, *S. epidermidis*, *S. saprophyticus*, and clinical *S. aureus* sp. A+, demonstrating that synergy can be strain-dependent. Similar observations have been reported in other studies involving biosynthesized SeNPs, where the effectiveness varied across different bacterial strains. For example, SeNPs synthesized from *Bacillus cereus* exhibited varying MIC values depending on the bacterial strain tested, highlighting the importance of understanding strain-specific responses to nanoparticle treatments (25).

In the case of *A. baumannii*, another pathogen of significant clinical concern, the combination treatment also showed enhanced effectiveness. The increase in growth inhibition zones underscores the potential for this combination to combat resistant strains. However, it is important to note that the clinical isolate of *A. baumannii* exhibited no growth inhibition when treated alone, indicating a level of resistance that emphasizes the challenges posed by multidrug-resistant pathogens that often possess intrinsic resistance mechanisms (26). This aligns with findings from other biosynthetic SeNP studies, where similar resistance patterns were observed in clinical isolates of *A.* species (27).

The results for *S. aureus* were somewhat less promising, as no significant increase in inhibition zones was observed among treatment groups. This suggests that while SeNPs and *C. vulgaris* CE may have individual effects on other strains, their combination does not provide additional benefits against this particular bacterium, indicating a lack of synergy. This finding is consistent with previous research on resistant strains, which has shown variable responses to SeNP treatments (28).

The ΣFIC values calculated throughout the study provide valuable insights into the interactions between the two treatments. These values indicate synergy for certain strains, including clinical isolates of *E. coli*, *A. baumannii*, *S. epidermidis*, *S. saprophyticus*, and clinical *S. aureus* sp. A+. A ΣFIC value lower than 1 suggests a strong synergistic interaction, highlighting the effectiveness of this combination against coagulase-negative staphylococci that harbor resistance genes. This finding is consistent with previous studies demonstrating that natural extracts can enhance the antimicrobial properties of nanoparticles (28).

While ampicillin-sensitive strains, such as *E. coli* and *S. aureus*, responded positively to SeNPs, the ampicillin-resistant strains did not exhibit the same synergistic effects. Specifically, the clinical strain of *E. coli* and the clinical strain of *A. baumannii* showed no synergistic response. However, another ampicillin-resistant strain of *A. baumannii* demonstrated notable responses to SeNPs, underscoring the complexity of resistance mechanisms and the need for further research (29).

Moreover, certain combinations of *C. vulgaris* CE and SeNPs resulted in significant reductions in both MIC and MBC values; however, the relationship between these two metrics varied across different bacterial strains. For instance, *E. coli* exhibited synergy when treated with both agents, as indicated by a substantial decrease in MIC values, suggesting enhanced bactericidal activity compared to individual treatments (28).

The combination of *C. vulgaris* CE and SeNPs demonstrates a synergistic antimicrobial effect against resistant bacterial strains through multiple mechanisms. Firstly, the phytochemicals in *C. vulgaris* CE induce oxidative stress, while SeNPs generate reactive oxygen species (ROS), overwhelming bacterial antioxidant defenses and promoting cell death. Secondly, *C. vulgaris* CE may inhibit bacterial drug efflux pumps, which increases the intracellular concentrations of both the extract and SeNPs, thereby enhancing efficacy against antibiotic-resistant strains. Additionally, the formulation of *C. vulgaris* CE with SeNPs improves the bioavailability and stability of bioactive compounds, ensuring sustained release and prolonged action against bacteria. These findings suggest that combining natural extracts with nanoparticles could be a promising strategy for addressing bacterial infections, particularly those caused by antibiotic-resistant strains, warranting further investigation into these mechanisms and potential clinical applications (30).

### 5.3. Cytotoxicity

Our findings from the MTT assay demonstrate that while both *C. vulgaris* CE and SeNPs decreased MCF-7 cell viability, their combination significantly enhances this effect, indicating a more pronounced cytotoxic effect when used together. Specifically, treatment with 5 mg/L of *C. vulgaris* CE resulted in an IC_50_ value of 4.38 mg/L, indicating potent anticancer activity that aligns with previous studies showing its effectiveness against the HeLa cell line. In comparison, doxorubicin (DOX), a widely used synthetic chemotherapeutic agent, exhibits a higher IC_50_ value of 13.3 mg/L, suggesting that *C. vulgaris* CE possesses greater potency in inhibiting cancer cell viability than DOX (17).

Furthermore, it is important to contextualize these findings against existing benchmarks for nanoparticle-based therapeutics. Studies have shown that the acceptable range for cytotoxicity in nanoparticle applications typically falls between 5 to 50 mg/L for effective therapeutic agents (18). In this context, the observed IC_50_ values for both *C. vulgaris* CE and SeNPs suggest that they are within an acceptable range for therapeutic applications. The viability observed with SeNPs aligns with previous studies that demonstrated their anticancer potential against colon, skin, and lung cancers without affecting human normal cells (18). The combined treatment of *C. vulgaris* CE and SeNPs resulted in a marked reduction in cell viability. This significant reduction is noteworthy, as it suggests a potential interaction between the two agents that could amplify their effects on target cells. The observed cytotoxicity in the combination treatment is consistent with findings from other researchers exploring the effects of nanoparticle combinations with natural extracts (19).

Given these promising results, further investigations are warranted to elucidate the underlying mechanisms responsible for the enhanced cytotoxic effects observed with the combination treatment. In summary, our results not only demonstrate the individual and combined efficacy of *C. vulgaris* CE and SeNPs against MCF-7 cells but also place these findings within the broader context of acceptable cytotoxicity ranges for nanoparticle-based therapeutics, thereby highlighting their potential for clinical applications. The translational potential of this article is discussed in the Supplementary File 1.

### 5.4. Conclusions

The isolation and characterization of the *Kocuria* strain Bm3 significantly enhance our understanding of microbial diversity within mangrove ecosystems, highlighting the potential applications of halotolerant bacteria in bioremediation and nanoparticle synthesis. Future research should prioritize elucidating the specific metabolic pathways involved in the synthesis of SeNPs and exploring their practical applications in both environmental and biomedical contexts. Zeta potential analysis is crucial for evaluating the colloidal stability and surface charge of nanoparticles; however, these measurements were not performed in the current study. Future investigations should incorporate zeta potential analysis to further elucidate the stability and therapeutic potential of SeNPs.

Our findings indicate that SeNPs significantly enhance the antibacterial activity of *C. vulgaris* CE against various bacterial strains, particularly non-clinical isolates. Notably, we observed varying degrees of resistance among clinical strains, underscoring the need for further exploration into these compounds' potential applications in developing more effective antimicrobial therapies. In terms of cytotoxicity, both *C. vulgaris* CE and SeNPs demonstrated enhanced cell viability when administered individually, with percentages of 49.75 ± 3.75% and 44.46 ± 4.26%, respectively. However, their combination resulted in a significant reduction in MCF-7 cell viability to 29.36 ± 2.64% (P < 0.001). This pronounced cytotoxic effect on cancer cells warrants further investigation into the underlying mechanisms responsible for this interaction.

Future studies should focus on mechanistic molecular investigations, including gene expression analyses such as quantitative PCR (qPCR) to assess the regulation of ROS genes and apoptosis markers in MCF-7 cells or relevant bacterial models. Additionally, comprehensive characterization of the chemical profile of *C. vulgaris* CE is essential. Investigating the specific contributions of total phenolics, proteins, and chlorophyll to the observed antibacterial and cytotoxic effects will enhance our understanding of their bioactive properties. Moreover, future research should explore the mechanisms underlying the interactions between SeNPs and *C. vulgaris* CE to optimize their therapeutic applications and develop effective strategies against antibiotic-resistant pathogens.

Expanding future tests to include a broader antimicrobial spectrum, such as fungi, gram-negative rods, and biofilm-forming bacteria, will validate the wider efficacy of these compounds. In vivo evaluations using appropriate animal models are also recommended to assess toxicity, bioavailability, and efficacy within complex biological systems. In summary, our research advances the field of microbial biotechnology while opening new avenues for therapeutic interventions against bacterial infections and cancer. This emphasizes the importance of continued exploration in these areas to fully realize the potential benefits of SeNPs and *C. vulgaris* CE in both environmental and medical applications.

## supplementary material

ijpr-24-1-164350.pdf

## Data Availability

The 16S rRNA gene sequence obtained in this study has been deposited in NCBI GenBank under accession number PV776680. Additionally, raw experimental data, including inhibition zone measurements and optical density (OD) values, are available upon request.
